# Sarcopenia, myosteatosis, and frailty parameters to predict adverse outcomes in patients undergoing emergency laparotomy: prospective observational multicentre cohort study

**DOI:** 10.1093/bjsopen/zraf016

**Published:** 2025-04-02

**Authors:** Brittany Park, Alain Vandal, Fraser Welsh, Tim Eglinton, Jonathan Koea, Ashish Taneja, Ahmed Barazanchi, Andrew G Hill, Andrew D MacCormick

**Affiliations:** Faculty of Medical and Health Sciences, The University of Auckland, Waipapa Taumata Rau, Auckland, Aotearoa New Zealand; Department of Surgery, Te Whatu Ora Counties Manukau, Auckland, Aotearoa New Zealand; Department of Statistics, The University of Auckland, Auckland, Aotearoa New Zealand; Faculty of Medical and Health Sciences, The University of Auckland, Waipapa Taumata Rau, Auckland, Aotearoa New Zealand; Faculty of Medical and Health Sciences, University of Otago, Christchurch, Aotearoa New Zealand; Faculty of Medical and Health Sciences, The University of Auckland, Waipapa Taumata Rau, Auckland, Aotearoa New Zealand; Faculty of Medical and Health Sciences, The University of Auckland, Waipapa Taumata Rau, Auckland, Aotearoa New Zealand; Department of Surgery, Auckland City Hospital, Te Whatu Ora, Auckland, Aotearoa New Zealand; Faculty of Medical and Health Sciences, The University of Auckland, Waipapa Taumata Rau, Auckland, Aotearoa New Zealand; Faculty of Medical and Health Sciences, The University of Auckland, Waipapa Taumata Rau, Auckland, Aotearoa New Zealand; Department of Surgery, Te Whatu Ora Counties Manukau, Auckland, Aotearoa New Zealand; Faculty of Medical and Health Sciences, The University of Auckland, Waipapa Taumata Rau, Auckland, Aotearoa New Zealand; Department of Surgery, Te Whatu Ora Counties Manukau, Auckland, Aotearoa New Zealand

## Abstract

**Background:**

Functional compromise contributes significantly to adverse outcomes after emergency laparotomy. Sarcopenia, defined as reduced muscle strength and muscle quantity, has been seldom assessed in patients undergoing emergency laparotomy. The aim of this study was to examine functional compromise in emergency laparotomy using sarcopenia, myosteatosis, and frailty parameters and evaluate impacts on functional and patient-centred outcomes.

**Methods:**

Patients aged greater than or equal to 55 years who underwent emergency laparotomy and preoperative computed tomography (CT) at two hospitals in New Zealand between February 2022 and October 2023 were included in a prospective database. Sarcopenia was measured using the SARC-F questionnaire, isokinetic dynamometry to measure hand grip strength, and skeletal muscle quantity according to CT. Myosteatosis was determined using CT and frailty was assessed using the Clinical Frailty Scale. Predictors for rehabilitation, days alive and out of hospital at 90 days, and risk of not returning home were analysed using relative risk and proportional means regression. Secondary outcomes were 3- and 6-month mortality and inpatient morbidity defined using the Clavien–Dindo classification.

**Results:**

A total of 101 patients undergoing emergency laparotomy during the study interval were analysed; 21.6% of participants had sarcopenia, 34.7% had myosteatosis, and 24.8% were living with frailty. Muscle strength parameters (low grip strength and a positive SARC-F questionnaire) had significant relationships with primary outcomes. Low grip strength (less than 27 kg for male patients and less than 16 kg for female patients) was most significant for risk of admission for rehabilitation (adjusted risk ratio 5.48 (95% c.i. 2.03 to 14.82)). A positive SARC-F questionnaire (an overall score of greater than or equal to 4 out of 10) was most significant for not returning home (adjusted risk ratio 8.26 (95% c.i. 1.81 to 37.76)). Isolated low muscle quantity (less than 52.4 cm^2^/m^2^ for male patients and less than 38.5 cm^2^/m^2^ for female patients) demonstrated no relationship. Being frail was most significant for a reduced number of days alive and out of hospital at 90 days (−13.4% compared with non-frail participants (95% c.i. −24.3% to −0.8%)). Sarcopenia and low grip strength were the only parameters to demonstrate a relationship with 3- and 6-month mortality.

**Conclusion:**

Sarcopenia and frailty parameters are major determinants of functional compromise and predict adverse outcomes after emergency laparotomy. Muscle strength is more important than mass, and measurable without imaging, streamlining its clinical application.

## Teaser

This study determined that sarcopenia (incorporating muscle strength parameters), myosteatosis, and frailty are major determinants of functional compromise, feasible to measure before surgery, and predict adverse patient-centred and functional outcomes for older patients after emergency laparotomy. Muscle strength is more important than mass, and measurable without imaging, streamlining its clinical application.

## Introduction

Preoperative assessment in emergency laparotomy (EL) is challenging with the acutely unwell surgical patient and a time-constrained setting^1^. A surgeon’s traditional clinical judgment or ‘end of the bed’ assessment has been shown to correctly identify only a small number of very high-risk patients^2,3^. Prospective candidates for EL are older, more co-morbid, and frailer, increasing the costs to the healthcare system^4^. Functional compromise contributes significantly to adverse outcomes in older patients and may provide more important information in a preoperative risk assessment than age^5^. The best way to assess functional compromise and resulting impacts on outcomes after EL are yet to be determined, whereas other indicators for frailty patients have been extensively investigated.

Sarcopenia or ‘muscle failure’ contributes to functional compromise^6^. This may be age-related or secondary to systemic processes such as malignancy^7^. A ‘strength, assistance with walking, rising from a chair, climbing stairs, and falls’ (‘SARC-F’) screening questionnaire has demonstrated high specificity to predict subsequent low muscle strength and is recommended as a preliminary way to identify individuals with probable sarcopenia^8,9^. Historically, low muscle quantity was used as an isolated parameter to define sarcopenia—attractive to clinicians because it may be captured as low skeletal muscle mass (skeletal muscle index (SMI)) using abdominal computed tomography (CT), which is routinely performed in the preoperative diagnostic workup^10^. The European and Asian Working Groups’ definition for sarcopenia was recently updated to place increasing emphasis on the importance of reduced muscle strength, with additional low muscle quantity or quality^6,11^. In the elective setting, sarcopenia can be feasibly measured in this way and has been correlated with morbidity and mortality up to 3 months after major abdominal surgery^12^. The impact of sarcopenia using the updated definition in populations undergoing EL, with the use of isokinetic dynamometry to measure muscle strength, has not been assessed.

Myosteatosis or skeletal muscle fat infiltration is a further body composition measure of skeletal muscle radiation attenuation (SM-RA)—representing loss of muscle quality and resulting loss of strength and function^13^. Visceral and intramuscular adipose indices may also be measured from CT imaging and have demonstrated an inverse relationship with sarcopenia^10^. Frailty may be characterized as a cumulative decline in multiple body systems or functions over a lifetime^14^. Recently, the Clinical Frailty Scale (CFS) was recommended by the National Emergency Laparotomy Audit (NELA) as the most appropriate tool for frailty assessment in patients undergoing EL^4,15^. Determination of CFS may be performed as a short preoperative clinical assessment under time constraints^15^. Frailty, sarcopenia, and myosteatosis have overlapping components and cumulatively contribute to an older individual’s functional compromise^16^.

The aim of this prospective multicentre cohort study was to examine functional compromise in older patients undergoing EL using parameters for sarcopenia, myosteatosis, and frailty, and to evaluate their impacts on functional and patient-centred outcomes. The secondary aim was to assess morbidity and mortality outcomes.

## Methods

This was a prospective multicentre cohort study designed following the STROBE statement guidelines^17^. Ethical approval was obtained from the New Zealand Health and Disability Ethics Committee (reference 17/STH/87) with local approval at each hospital site. This study was not pre-registered.

### Patients and data

From February 2022 to October 2023, patients aged 55 years or older presenting acutely and undergoing EL at two public tertiary hospitals (Auckland Hospital in Central Auckland and Middlemore Hospital in South Auckland) were screened for inclusion. To be included, patients were required to have undergone abdominal CT as part of their preoperative assessment. EL was defined, in accordance with the NELA definition, as acute laparotomy or a major laparoscopic or laparoscopically assisted procedure performed for any indication, including reoperation for a complication^4^. Exclusion criteria were age less than 55 years, elective operation, appendectomy, hernia repair without bowel resection, laparotomy for trauma, vascular, biliary, gynaecological, urological, and transplant procedures. Written informed consent was obtained from eligible patients. Participants with cognitive impairment or perioperative delirium were included to a limited extent and parts of the functional compromise assessment were only conducted where clinically appropriate.

The following variables were captured: participant demographics (age, sex, ethnicity, and hospital allocation); operative indication and approach; presence or absence of cancer pathology (primary malignancy *versus* disseminated disease); albumin level; cardiopulmonary and diabetes co-morbidities; body mass index, weight, and height; and American Society of Anesthesiologists (ASA) classification. Sarcopenia, myosteatosis, adiposity, and frailty measures were recorded as detailed below. The participant’s residence before admission, dependence level (independent, partially dependent, or totally dependent), required level of care, and number of falls (0, 1, or greater than or equal to 2) in the preceding year were recorded before surgery. The level of care was recorded as none required, ‘home help’ required (assistance with household chores, such as vacuuming and shopping), or ‘personal care’ required (assistance with daily care, such as showering). The residence from which a patient was admitted was recorded as their own home, a support unit (assisted living, such as a retirement village), a rest home (a retirement home with an increased level of care), or a private hospital/dementia unit (for patients living in a facility with full-time care requirements). Participants were followed for up to 6 months.

### Body composition analysis

CT images were anonymized and centralized using a secure image exchange portal after undergoing quality checking. Cross-sectional slices at the level of the superior endplate of the third lumbar vertebrae (L3) were identified manually. The Digital Imaging and Communications in Medicine images were extracted from local hospital systems. Anonymized L3 images were transferred to SliceOmatic software (TomoVision, Magog, Quebec, Canada; version 5.0) on a secure device and analysed by two researchers/doctors blinded with regard to participant and outcome data and who had undergone software training.

### Sarcopenia analysis

For skeletal muscle mass analyses, the cross-sectional area (cm^2^) at L3 of skeletal muscle (−29 to 150 Hounsfield units (HU)) was calculated using predefined HU ranges. The threshold for low skeletal muscle was defined as below the lowest sex-specific tertile, based on previously validated methods^10^. Cross-sectional areas were adjusted for height squared to calculate SMI. Based on previously validated thresholds, reduced SMI was defined based on a cut-off value of 52.4 cm^2^/m^2^ for male patients and 38.5 cm^2^/m^2^ for female patients^18^.

For skeletal muscle function analyses, isokinetic dynamometry using a calibrated Jamar handheld dynamometer^19^ was used to assess maximum voluntary hand grip strength (HGS) in kilograms either before surgery or at least a minimum of 24 h after surgery to allow time for recovery. The highest value of three attempts using the participant’s dominant hand was taken as the result. Reduced HGS was defined as less than 27 kg for men and less than 16 kg for women, based on current European and Asian Working Group definitions^6,11^. ‘Combined sarcopenia’ was defined as reduced muscle strength (low HGS) with the addition of low muscle quantity (low SMI) in accordance with the updated European and Asian Working Groups’ definition^6,11^.

In addition, the SARC-F questionnaire was utilized. Each self-reported parameter received a score from zero to two, with a maximum possible score of ten^8,9^. An overall score of greater than or equal to four was considered a positive screening questionnaire based on the predefined threshold^8,9^.

### Myosteatosis and adipose tissue analysis

Mean SM-RA was assessed by calculating the mean HU value of the total muscle area within the specified skeletal muscle range (−29 to 150 HU). This excluded intramuscular adipose tissue (IMAT). Predefined HU ranges were used to measure the cross-sectional areas (cm^2^) for visceral adipose tissue (VAT; −150 to −50 HU) and IMAT (−190 to −30 HU). VAT was further adjusted for height squared (as with SMI) to calculate the VAT index (VATI). Thresholds were calculated based on sex-specific tertiles, consistent with previous validated methods^12^. Myosteatosis was defined as SM-RA within the lowest sex-specific tertile. ‘High’ VATI and IMAT measures were defined as values within the highest respective tertiles.

### Frailty analysis

Frailty was assessed using the CFS. This was measured by the study recruiter at the time of recruitment (research clinician or research nurse). Using the updated Likert scale (ranging from 1 to 9), participants scoring greater than or equal to five (‘mildly frail’ to ‘terminally ill’) were classified as ‘frail’ and those scoring less than five (‘very fit’ to ‘vulnerable’) were considered ‘non-frail’^14^.

### Outcomes of interest

Primary outcomes were risk of admission for rehabilitation, the risk of not returning home, and whether participants from home returned home and days alive and out of hospital at 90 days (90 DAOH). Secondary outcomes were major complications measured using the Clavien–Dindo classification^20^, duration of hospital stay, and mortality at 3 and 6 months.

### Statistics

Statistical methodology was developed with a senior biostatistician. All analyses were conducted using R (R Foundation for Statistical Computing, Vienna, Austria; version 3.6.3).

Functional compromise parameters are presented as sex-specific medians and interquartile ranges. Thresholds are presented as predefined values for HGS, SMI, SARC-F, and CFS, with the corresponding number and percentage of participants. Thresholds are presented as the lowest tertile value for SM-RA and highest tertile values for VATI and IMAT. Frequencies of binary outcomes are presented as absolute numbers and percentages. Categorical outcomes were analysed using Fisher’s exact test to account for small sample sizes, whereas continuous variables were compared using the Mann–Whitney *U* test due to non-normal data distributions. Results are reported as *P* values to assess statistical significance between groups. Continuous data are presented as means and standard deviations. A composite outcome was created for participants admitted from home who did not return home, either because they died as an inpatient or were discharged to a facility with an increased level of care. The outcome of ‘90 DAOH’ was re-scaled between zero and one for analysis.

Simple and multiple regression analysis was conducted for the following functional compromise parameters: ‘combined sarcopenia’, ‘low HGS’, ‘low SMI’, and ‘SARC-F positive’ to represent sarcopenia and ‘low SM-RA’ to represent myosteatosis, based on the above-defined thresholds, and a ‘CFS greater than or equal to five’ to represent frailty—to assess which of these parameters independently predicted primary and secondary outcomes. Multiple regression was conducted using a direct acyclic graph (DAG) (*Figs. S1–S3*) to select potential confounders. Age, sex, and ethnicity were included in the model based on pre-existing literature and clinical significance. Variables that were identified as possible confounders from the DAG due to a causal relationship with functional compromise were then selected for the model based on the significance of their relationship with each outcome (defined by a 2-tailed *P* < 0.050). Risk ratios (RRs) and 95% confidence intervals were calculated using binomial regression with log link for binary outcomes. For continuous outcomes, 90 DAOH was analysed using a quasi-binomial model with log link (as percentage differences in mean 90 DAOH from the reference category (0% change or no difference in 90 DAOH) with associated 95% confidence intervals) and duration of hospital stay was analysed using a quasi-likelihood model with quadratic variance function and log link with similarly interpretable outcomes. Hospital allocation was fitted in simple and multiple regression models as a random effect to account for variability. In instances where a low number of outcome events led to infinite confidence intervals for a group on simple regression analysis, this parameter was then excluded from further analysis to mitigate the issue of complete separation and ensure the stability of the model.

## Results

A total of 102 participants were included in the prospective database; one participant did not undergo CT imaging and, therefore, 101 participants were included in the final analysis. Of these, four participants did not have HGS measured and eight participants did not complete a SARC-F questionnaire. Surgery was most performed as an open procedure (89.1%), with bowel obstruction as the most common indication (61.4% of participants) (*Table 1*). Most participants were European, aged 65–80 years, and had an ASA grade greater than II. Primary malignancy was found in 11.9% of participants, with disseminated disease in a further 11.9%.

**Table 1:** Participant demographics; *n* = 101

	Value
**Site**	
South Auckland	61 (60.4)
Central Auckland	40 (39.6)
**Ethnicity**	
European	68 (67.3)
Asian	10 (9.9)
Māori	12 (11.9)
Pacific	9 (8.9)
Other	2 (2.0)
**Sex**	
Male	48 (47.5)
Female	53 (52.5)
**Age group (years)**	
55–64	27 (26.7)
65–80	56 (55.4)
>80	18 (17.8)
Age (years), mean(s.d.)	72(8.9)
BMI (kg/m^2^), mean(s.d.)	27.3(8.6)
Cardiopulmonary disease	20 (19.8)
Diabetes	16 (15.8)
Hypoalbuminaemia	42 (41.6)
**ASA grade**	
I–II	33 (32.7)
>II	68 (67.3)
**Operative indication**	
Obstruction	62 (61.4)
Perforation	17 (16.8)
Ischaemia	9 (8.9)
Wound dehiscence	6 (5.9)
Volvulus	2 (2.0)
Anastomotic leak	1 (1.0)
Bleeding	2 (2.0)
Intussusception	1 (1.0)
Peritonism	1 (1.0)
**Cancer**	
No	77 (76.2)
Primary	12 (11.9)
Disseminated	12 (11.9)
**Operative approach**	
Laparoscopic	6 (5.9)
Open	90 (89.1)
Laparoscopic converted to open	5 (5.0)

Values are *n* (%) unless otherwise indicated. BNI, body mass index.; ASA, American Society of Anesthesiologists.

### Functional compromise parameters in older emergency laparotomy patients

**Fig. 1:**
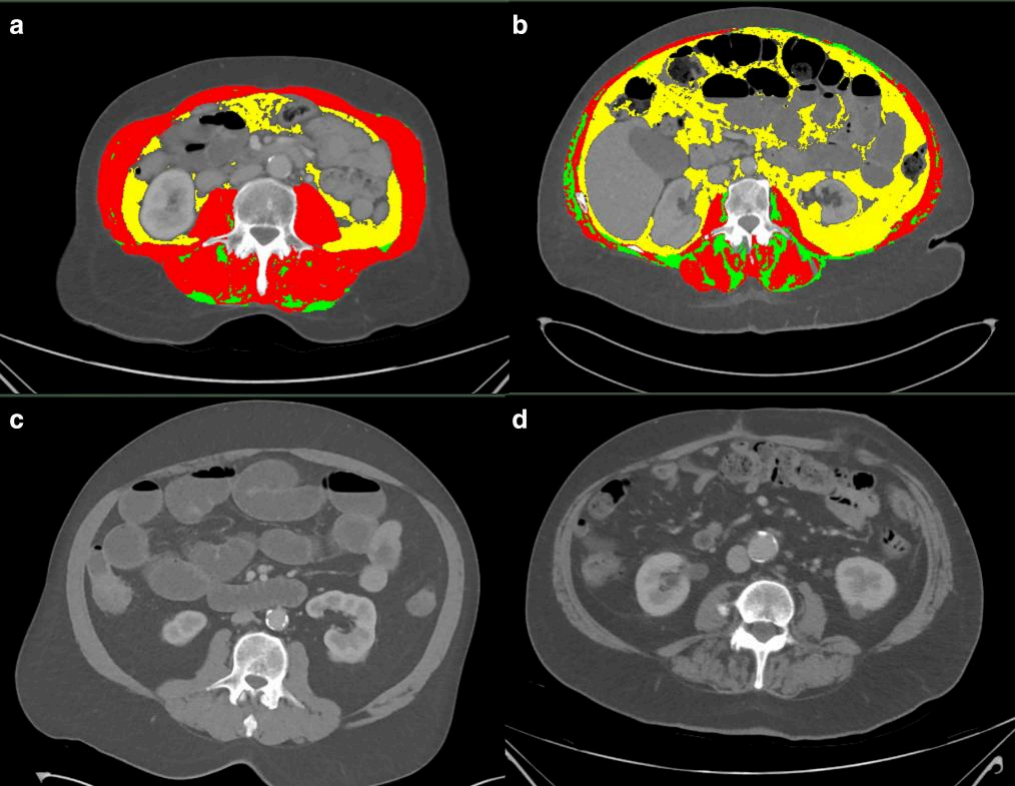
Comparison of normal *versus* reduced skeletal muscle mass and normal *versus* reduced skeletal muscle radiation attenuation **a** Female participant with normal skeletal muscle mass (red = skeletal muscle, yellow = visceral adipose tissue, and green = intramuscular adipose tissue). **b** Female participant with reduced skeletal muscle mass (red = skeletal muscle, yellow = visceral adipose tissue, and green = intramuscular adipose tissue). **c** Male participant with normal skeletal muscle radiation attenuation. **d** Male participant with reduced skeletal muscle radiation attenuation.

Functional compromise parameters with median values and thresholds in older participants undergoing EL are presented in *Table 2*. Although the rate of a positive SARC-F questionnaire was almost two-fold higher in female patients compared with male patients, the prevalence of combined sarcopenia was higher in male patients (25.0% compared with 17.0% of female patients). Reduced muscle strength (HGS) was similar between groups (present in 35.4% of male patients and 30.2% of female patients) and a larger proportion of male patients had reduced muscle quantity (68.8% *versus* 43.4%). Conversely, frailty prevalence was higher in women (28.3% with a CFS greater than or equal to 5 *versus* 20.8% of men). While the median SM-RA was similar between sexes, the threshold for the presence of myosteatosis was higher in male patients (28.0 *versus* 22.1 HU). Regarding adiposity, median VATI measurements and thresholds were higher in male patients, whereas IMAT values were higher in female patients. A comparison of normal *versus* reduced skeletal muscle mass and normal *versus* reduced SM-RA is presented in *Fig. 1* (SliceOmatic software (TomoVision, Magog, Quebec, Canada; version 5.0)).

**Table 2:** Physiological parameters in emergency laparotomy patients

	Male			Female		
	Median (i.q.r.)	Threshold	*n* (%)*	Median (i.q.r.)	Threshold	*n* (%)*
**Sarcopenia measurements**						
Skeletal muscle (cm^2^)	145.8 (119.1–179.4)	115.9	–	103.7 (90.4–140.9)	81.7	–
Skeletal muscle index (cm^2^/m^2^)	42.8 (35.6–54.4)	52.4	33 (68.8)	39.2 (32.7–50.8)	38.5	23 (43.4)
Hand grip strength (kg)	29.4 (24.1–38.1)	27	17 (35.4)	18.0 (15.0–21.7)	16	16 (30.2)
SARC-F questionnaire	0 (0–2.5)	4	8 (16.7)	2 (0–4)	4	16 (30.2)
Combined sarcopenia	–	<52.4 cm^2^/m^2^, <27 kg	12 (25.0)	–	<38.5 cm^2^/m^2^, <16 kg	9 (17.0)
**Myosteatosis measurement**						
Skeletal muscle radiation attenuation (Hounsfield units)	31.0 (26.6–38.6)	28.0	–	28.4 (22.1–35.6)	22.1	–
**Frailty measurement**						
Clinical Frailty Scale	3 (1–4)	5	10 (20.8)	3 (2–5)	5	15 (28.3)
**Adipose measurements**						
Visceral adipose tissue index (cm^2^/m^2^)	56.2 (22.3–66.9)	66.2	–	23.5 (7.1–46.6)	33.5	–
Intermuscular adipose tissue (cm^2^)	17.3 (8.9–24.9)	18.0	–	20.1 (10.2–29.1)	24.7	–

*Number and percentage of patients below the threshold value. All thresholds were predefined, except those for combined sarcopenia. Missing values: male patients, five missing SARC-F scores; and female patients, three missing SARC-F scores and four missing hand grip strength scores (impacting combined sarcopenia values). Combined sarcopenia was defined as meeting both criteria: skeletal muscle index below the threshold and hand grip strength below the threshold. i.q.r., interquartile range; SARC-F, ‘strength, assistance with walking, rising from a chair, climbing stairs, and falls’.

### Pre-admission baseline in functional compromise groups

Overall, 21.6% of participants had sarcopenia (using the combined definition), 34.7% had myosteatosis, and 24.8% were living with frailty. *Table 3* demonstrates the level of dependence, number of falls in the preceding year, required care, and residence before admission for participants living with and without these conditions. Participants living with each condition demonstrated a higher dependency status, number of falls, and care level than those in comparison groups. Of frail participants, 40.0% were partially or fully dependent compared with just 5.3% of non-frail participants before admission, and 32.0% required home help or personal care (compared with 5.2%). In the sarcopenia group, 38.1% of participants had greater than or equal to one fall, compared with 11.8% of the non-sarcopenia group.

**Table 3:** Pre-admission baseline in functional compromise groups

	Sarcopenia		Myosteatosis		Frailty	
	Yes (*n* = 21)	No (*n* = 76)	Yes (*n* = 35)	No (*n* = 66)	Yes (*n* = 25)	No (*n* = 76)
**Dependence**						
Independent	15 (71.4)	69 (90.8)	26 (74.3)	61 (92.4)	15 (60.0)	72 (94.7)
Partially dependent	5 (23.8)	6 (7.9)	7 (20.0)	5 (7.6)	8 (32.0)	4 (5.3)
Fully dependent	1 (4.8)	1 (1.3)	2 (5.7)	0 (0.0)	2 (8.0)	0 (0.0)
**Care level**						
None	16 (76.2)	70 (92.1)	29 (82.9)	60 (90.9)	17 (68.0)	72 (94.7)
Home help	4 (19.0)	4 (5.3)	4 (11.4)	4 (6.1)	6 (24.0)	2 (2.6)
Personal care	1 (4.8)	2 (2.6)	2 (5.7)	2 (3.0)	2 (8.0)	2 (2.6)
**Residence admitted from**						
Own home	19 (90.5)	74 (97.4)	32 (91.4)	64 (97.0)	22 (88.0)	74 (97.4)
Support unit	1 (4.8)	2 (2.6)	2 (5.7)	2 (3.0)	2 (8.0)	2 (2.6)
Rest home	1 (4.8)	0 (0.0)	1 (2.9)	0 (0.0)	1 (4.0)	0 (0.0)
Private hospital/dementia unit	0 (0.0)	0 (0.0)	0 (0.0)	0 (0.0)	0 (0.0)	0 (0.0)
**Falls in the last year**						
0	13 (61.9)	67 (88.2)	27 (77.1)	57 (86.4)	17 (68.0)	67 (88.2)
1	5 (23.8)	3 (3.9)	4 (11.4)	4 (6.1)	3 (12.0)	5 (6.6)
≥2	3 (14.3)	6 (7.9)	4 (11.4)	5 (7.6)	5 (20.0)	4 (5.3)

Values are *n* (%).

### Primary outcomes: rehabilitation and patients not returning home

A requirement for admission to a rehabilitation unit after surgical admission was over three times higher for participants in the sarcopenia and myosteatosis groups (38.1% of the sarcopenia group (*P* < 0.050) and 31.4% of the myosteatosis group (*P* < 0.050)) (*Table 4*). On univariable regression analysis, all functional compromise parameters apart from a positive SARC-F questionnaire and low SMI were significant predictors for admission for rehabilitation (*Table 5*). Age greater than 80 years and an ASA grade greater than II were significant confounders and subsequently adjusted for with sex and ethnicity. On multiple regression analysis, low HGS demonstrated the strongest relationship with admission for rehabilitation (adjusted RR (aRR) 5.48 (95% c.i. 2.03 to 14.82), *P* < 0.001), followed by combined sarcopenia (aRR 3.09 (95% c.i. 1.62 to 5.92), *P* < 0.001) and a CFS greater than or equal to five (aRR 2.14 (95% c.i. 1.04 to 4.44), *P* = 0.040). Low SM-RA was no longer significant (*P* = 0.063).

**Table 4:** Primary and secondary outcomes by functional compromise groups after emergency laparotomy

	Sarcopenia			Myosteatosis			Frailty		
	Yes (*n* = 21)	No (*n* = 76)	*P*	Yes (*n* = 35)	No (*n* = 66)	*P*	Yes (*n* = 25)	No (*n* = 76)	*P*
**Primary outcomes**									
90 DAOH, median (i.q.r.)	69.0 (22.0)	79.0 (13.0)	<0.001	71.0 (23.0)	80.0 (13.0)	<0.001	70.0 (23.0)	79.0 (14.0.0)	<0.001
Rehabilitation	8 (38.1)	8 (10.5)	0.006	11 (31.4)	5 (7.6)	0.003	7 (28.0)	9 (11.8)	0.066
From home, but did not return home	4 (19.0)	5 (6.6)	0.099	7 (20.0)	2 (3.0)	0.008	6 (24.0)	3 (3.9)	0.007
**Secondary outcomes**									
Mortality									
Inpatient mortality	2 (9.5)	0 (0.0)	0.031	2 (5.7)	0 (0.0)	0.608	1 (4.0)	1 (1.3)	0.998
90-day mortality	3 (14.3)	1 (1.3)	0.231	2 (5.7)	2 (3.0)	0.901	1 (4.0)	3 (3.9)	0.559
Six-month mortality	3 (14.3)	2 (2.6)	0.870	2 (5.7)	3 (4.5)	0.875	1 (4.0)	4 (5.3)	0.804
Major complications	4 (19.0)	21 (27.6)	0.010	11 (31.4)	15 (22.7)	0.138	7 (28.0)	19 (25.0)	0.027
Duration of hospital stay, median (i.q.r.)	13.0 (11.0)	10.0 (12.3)	<0.001	16.0 (16.0)	9.0 (9.0)	<0.001	17.0 (17.0)	10.0 (9.0)	<0.001
Discharge destination if admitted from home*									
Own home	15 (78.9)	69 (93.2)	0.032	26 (81.3)	62 (96.9)	0.010	17 (77.3)	71 (95.9)	0.003
Support unit	1 (5.3)	3 (4.1)	0.901	3 (9.4)	1 (1.6)	0.119	2 (9.1)	2 (2.7)	0.255
Rest home	0 (0.0)	0 (0.0)	NA	0 (0.0)	0 (0.0)	NA	0 (0.0)	0 (0.0)	NA
Private hospital/dementia unit	1 (5.3)	2 (2.7)	0.523	2 (6.3)	1 (1.6)	0.275	3 (13.6)	0 (0.0)	0.014

Values are *n* (%) unless otherwise indicated. *Missing data: four patients for sarcopenia group (missing hand grip strength). 90 DAOH, days alive and out of hospital at 90 days; i.q.r., interquartile range; NA, not applicable.

**Table 5:** Relative risk regression analysis for admission for rehabilitation

	Simple regression analysis		Multiple regression analysis	
	RR (95% c.i.)	*P*	RR (95% c.i.)	*P*
**Combined sarcopenia**				
No	Reference		Reference	
Yes	4.00 (1.72,9.28)	<0.001	3.09 (1.62,5.92)	<0.001
**Low HGS**				
No	Reference		Reference	
Yes	6.22 (2.18,17.80)	<0.001	5.48 (2.03,14.80)	<0.001
**Low SMI**				
No	Reference		Reference	
Yes	1.07 (0.43,2.66)	0.900	0.72 (0.35,1.46)	0.360
**SARC-F positive**				
No	Reference		Reference	
Yes	1.91 (0.70,5.25)	0.210	1.94 (0.69,5.42)	0.210
**Low SM-RA**				
No	Reference		Reference	
Yes	4.51(1.70,12.00)	0.003	2.27 (0.96,5.38)	0.063
**Clinical Frailty Scale ≥5**				
No	Reference		Reference	
Yes	2.56 (10.60,6.20)	0.037	2.14 (1.04,4.44)	0.040
**Age (years)**				
≤80	Reference			
>80	2.89 (1.21,6.89)	0.016	–	–
**BMI (kg/m^2^)**				
≥18.5 to <25	Reference			
≥25	0.33 (0.13,0.88)	0.062	–	–
<18.5	0.74 (0.11,4.94)	0.760	–	–
**Diabetes**				
No	Reference			
Yes	0.73 (0.18,2.94)	0.660	–	–
**Cardiopulmonary disease**				
No	Reference			
Yes	1.92 (0.76,4.87)	0.170	–	–
**Hypoalbuminaemia**				
No	Reference			
Yes	1.94 (0.77,4.85)	0.160	–	–
**Cancer**				
No	Reference			
Primary	1.03 (0.27,4.02)	0.960	–	–
Disseminated	0.48 (0.07,3.36)	0.460	–	–
**ASA grade**				
I–II	Reference			
>II	7.75 (1.07,56.24)	0.043	–	–

RR, risk ratio; HGS, hand grip strength; SMI, skeletal muscle index; SM-RA, skeletal muscle radiation attenuation; BMI, body mass index; ASA, American Society of Anesthesiologists.

Two participants died as inpatients (*Table 4*). A further 7.3% were discharged to a facility with an increased level of care, having lived in their own home before surgery. On regression analysis, low SM-RA, a CFS greater than or equal to five, low HGS, and a positive SARC-F questionnaire were significant predictors for participants ‘not returning home’ (*Table 6*). After multiple regression adjustment, a positive SARC-F questionnaire demonstrated the highest RR for not returning home (aRR 8.26 (95% c.i. 1.81 to 37.76), *P* = 0.007), followed by a CFS greater than or equal to five (aRR 6.38 (95% c.i. 1.89 to 21.57), *P* = 0.003), low SM-RA (aRR 4.66 (95% c.i. 1.12 to 19.39), *P* = 0.034), and low HGS (aRR 3.66 (95% c.i. 1.05 to 12.74), *P* = 0.042).

**Table 6:** Relative risk regression analysis for not returning home for patients admitted from home

	Simple regression analysis		Multiple regression analysis	
	RR (95% c.i.)	*P*	RR (95% c.i.)	*P*
**Combined sarcopenia**				
No	Reference			
Yes	3.00 (0.89,10.10)	0.076	2.14 (0.72,6.35)	0.170
**Low HGS**				
No	Reference			
Yes	4.24 (1.13,15.80)	0.032	3.66 (1.05,12.70)	0.042
**Low SMI**				
No	Reference			
Yes	0.98 (0.28,3.42)	0.970	0.57 (0.22,1.44)	0.230
**SARC-F positive**				
No	Reference			
Yes	5.13 (1.21,21.80)	0.027	8.26 (1.81,37.80)	0.007
**Low SM-RA**				
No	Reference			
Yes	7.24 (1.59,32.90)	0.010	4.66 (1.12,19.40)	0.034
**Clinical Frailty Scale ≥5**				
No	Reference			
Yes	7.49 (2.05,27.30)	0.002	6.38 (1.89,21.60)	0.003
**Age (years)**				
≤80	Reference			
>80	2.59 (0.73,9.19)	0.140	–	–
**BMI (kg/m^2^)**				
<25	Reference			
≥25	0.94 (0.27,3.29)	0.930	–	–
**Cardiopulmonary disease**				
No	Reference			
Yes	1.09 (0.25,4.86)	0.910	–	–
**Hypoalbuminaemia**				
No	Reference			
Yes	2.99 (0.79,11.40)	0.110	–	–
**Cancer**				
No	Reference			
Primary	0.91 (0.12,6.78)	0.930	–	–

Excluded: ASA grade greater than II, diabetes, and disseminated cancer. RR, risk ratio; HGS, hand grip strength; SMI, skeletal muscle index; SM-RA, skeletal muscle radiation attenuation; BMI, body mass index.

A reduced median 90 DAOH was demonstrated for each of the participant groups living with sarcopenia, myosteatosis, and frailty (*Table 4*). Simple regression analysis revealed that low HGS and low SM-RA were associated with a reduction in 90 DAOH (*Table 7*). Hypoalbuminaemia and an ASA grade greater than II were significant confounders and entered into the model along with age, sex, and ethnicity. After adjustment, a CFS greater than or equal to five became significantly correlated with a reduction in 90 DAOH (−13.4% (95% c.i. −24.3% to −0.8%), *P* = 0.040), followed by low HGS (−12.6% (95% c.i. −23.5% to −0.2%), *P* = 0.050). Low SM-RA was no longer significant after adjustment (*P* = 0.073).

**Table 7:** Proportional means (bounded) regression analysis for days alive and out of hospital at 90 days

	Simple regression analysis		Multiple regression analysis	
	PD (95% c.i.)	*P*	PD (95% c.i.)	*P*
**Combined sarcopenia**				
No	Reference			
Yes	−15.6 (−29.8,+1.3)	0.072	−10.7 (−24.4,+5.4)	0.180
**Low HGS**				
No	Reference			
Yes	−15.0 (−26.5,−1.7)	0.031	−12.6 (−23.5,−0.2)	0.0496
**Low SMI**				
No	Reference			
Yes	−3.9 (−14.9,+8.5)	0.520	3.9 (−8.0,+17.4)	0.540
**SARC-F positive**				
No	Reference			
Yes	−15.8 (−29.3,+0.3)	0.058	−13.2 (−25.9,+1.6)	0.082
**Low SM-RA**				
No	Reference			
Yes	−14.3 (−25.4,−1.6)	0.031	−11.6 (−22.6,+1.0)	0.073
**Clinical Frailty Scale ≥5**				
No	Reference			
Yes	−14.5 (−27.1,+0.4)	0.059	−13.4 (−24.3,−0.8)	0.040
**Age (years)**				
≤80	Reference			
>80	−4.3 (−19.3,+13.6)	0.620	–	–
**BMI (kg/m^2^)**				
≥18.5 to <25	Reference			
≥25	+10.3 (−2.9,+25.2)	0.130	–	–
<18.5	+5.5 (−13.5,+28.6)	0.600	–	–
**Diabetes**				
No	Reference			
Yes	+9.2 (−5.0,+25.6)	0.220	–	–
**Cardiopulmonary disease**				
No	Reference			
Yes	−12.8 (−27.0,+4.1)	0.130	–	–
**Hypoalbuminaemia**				
No	Reference			
Yes	−14.6 (−24.7,−3.1)	0.016	–	–
**Cancer**				
No	Reference			
Primary	+1.0 (−1.6,+21.0)	0.910	–	–
Disseminated	−9.6 (−27.6,+12.8)	0.370	–	–
**ASA grade**				
I–II	Reference			
>II	−12.4 (−22.0,−1.7)	0.027	–	–

PD, percentage difference; HGS, hand grip strength; SMI, skeletal muscle index; SM-RA, skeletal muscle radiation attenuation; BMI, body mass index.

### Secondary outcomes: duration of hospital stay, morbidity, and mortality

The duration of hospital stay was longer for participants in each of the functional compromise groups (*Table 4*). On linear regression analysis, a CFS greater than or equal to five, low SM-RA, and an ASA grade greater than II were correlated with an increased duration of hospital stay (*Table S1*). However, after adjustment, only a CFS greater than or equal to five remained a significant predictor for increased duration of hospital stay (+55.4% (95% c.i. +1.5% to +143.2%), *P* = 0.038).

By 90 days, three further participants had died (*Table 4*). On analysis, combined sarcopenia was the only parameter to demonstrate a relationship with 90-day mortality (*Table S2*). After adjustment for age, sex, and ethnicity, combined sarcopenia (aRR 8.65 (95% c.i. 1.13 to 66.10), *P* = 0.038) and low HGS (aRR 8.36 (95% c.i. 1.10 to 63.50), *P* = 0.040) both demonstrated a relationship with 90-day mortality. These relationships remained consistent for 6-month mortality, at which time only one further participant had died (*Table S3*). Of the participants, 25.7% experienced a major complication (Clavien–Dindo grade greater than or equal to III). A relationship between hypoalbuminaemia and major complications was demonstrated that was not shown with functional parameters (*Table S4*).

## Discussion

This study evaluates functional compromise using sarcopenia, myosteatosis, and frailty parameters in older patients undergoing EL and illustrates the relationships between these conditions and adverse outcomes after surgery.

Parameters associated with reduced muscle strength (isokinetic dynamometry, the SARC-F questionnaire, and myosteatosis) demonstrate consistent relationships with all primary outcomes in the analysis. This indicates that muscle strength is more important than mass in preoperative assessment of functional compromise. Combined sarcopenia directly correlates with participants being admitted for rehabilitation and dying within 3 and 6 months. Conversely, low muscle quantity shows no relationship with any outcome.

Inconsistency is prevalent in the literature regarding sarcopenia defined as low muscle quantity. A recent meta-analysis of 20 EL studies demonstrated that, despite a relationship with mortality up to 3 months after surgery, after multivariable adjustment this was no longer significant^21^. Despite increased efforts, heterogeneity in methods of quantifying muscle quantity and a historical lack of standardized thresholds that are applicable within ethnically diverse patient populations have made application difficult in the clinical setting^22^. The findings of the present study support investigation into the use of muscle strength parameters to define sarcopenia, without the use of muscle mass. Myosteatosis prevalence in the participants of the present study is similar to that in a large multicentre UK study (34.7% *versus* 33.6% respectively)^5^. Frailty prevalence is slightly higher than in other large national studies (24.8% *versus* 20.0% of ELF study participants in the UK and 10.6% of ELLSA study participants in Scotland)^23,24^. In these studies, independent relationships were demonstrated with myosteatosis, frailty, and postoperative complications and mortality^23,25^. Given the low number of deaths in the present study, further investigation into these relationships is recommended, as well as to confirm the relationship demonstrated with combined sarcopenia.

In the present study, 15.8% of patients were admitted for rehabilitation. Low HGS was most strongly correlated with this outcome, which is easy to determine in clinical practice. Older age was significant, emphasizing that this must still be taken into consideration when examining the relationship between functional compromise and risk of admission for rehabilitation. Albumin demonstrated a relationship with reduced 90 DAOH, as well as major complications. This biomarker, associated with malnutrition, is routinely measured in clinical practice and as part of comprehensive geriatric assessment (CGA)^26^. However, its use in the emergency setting to assess functional compromise may be unreliable, given that it is an acute phase protein that alters with certain pathologies^27^.

This study used the composite outcome of combined inpatient death and increased level of care as ‘risk of not returning home’. This was established from the authors’ previous qualitative work with patients undergoing EL who felt that living in a nursing home, always needing care, and not being able to get out of bed would be close to, the same as, or worse than death^28^. For some older patients, not returning to their home may be of equal weighting to dying from their operation and this knowledge could contribute to patient-centred decision-making. Most functional parameters were correlated with this outcome. Frailty demonstrated a relationship where age did not. Age and frailty status in the ELF study were significantly correlated with participants from all residences being discharged to a facility with an increased level of care, which occurred for 37% of their participants^29^. Similar to the findings of the present study, Carter *et al*.^29^ found frailty had a stronger predictive power than age.

The functional compromise parameters may be appropriately measured in the ‘real-time’ setting of EL. Most patients in high- and middle-income countries will have CT imaging before undergoing EL. In addition, software for body composition analysis and use of artificial intelligence are becoming readily available^30^. However, in resource-constrained countries with limited access to imaging, sarcopenia parameters focusing on muscle strength may be used as a reliable way of assessing functional compromise. Many of the tools do not require formal training and are easy to use and interpret by clinicians and others^12,23^. Formal recommendations and thresholds by NELA and sarcopenia working groups on how to define frailty and sarcopenia mean that uniform assessment is now achievable in the clinical setting, where historically these concepts had largely been reviewed retrospectively in academic works^4,6,11,15^. The lowest tertile thresholds to define myosteatosis in the present study (male threshold of 28.0 HU and female threshold of 22.1 HU) are similar to those in a large multicentre UK study (male threshold of 29.3 HU and female threshold of 24.2 HU)^5^. The authors recommend formalizing defined thresholds, to bring myosteatosis assessment into the clinical setting.

This study has several limitations. Recruitment was impacted by national isolation requirements during the COVID-19 pandemic. Additionally, the rarity of some outcome events such as mortality led to wide confidence intervals and the problem of complete separation, resulting in infinite confidence intervals for several potential confounders, which were then excluded from the model for multiple regression analysis to maintain stability and interpretability. A larger sample size would likely mitigate these issues. Regarding the chosen clinically relevant parameters, the authors assumed these to have a causal rather than consequential relationship with functional compromise, although overlap may exist.

Overall, sarcopenia, myosteatosis, and frailty parameters are major determinants of functional compromise and predict adverse outcomes for older patients after EL. Muscle strength is more important than mass in this assessment and can be measured without imaging, streamlining its clinical application. Detecting functional compromise may enhance risk assessment to inform patient-centred decision-making and tailoring of perioperative and postoperative care bundles—ultimately improving outcomes.

## Supplementary Material

zraf016_Supplementary_Data

## Data Availability

A.V. is an expert biostatistician who verified the underlying data and analysis. The data are not publicly available, but will be available upon direct request to the corresponding author.
