# Supplementary material for: Sarcopenia, myosteatosis, and frailty parameters to predict adverse outcomes in patients undergoing emergency laparotomy: prospective observational multicentre cohort study
Source: BJS Open. 2025 Apr 2;9(2):zraf016. doi: 10.1093/bjsopen/zraf016 (PMC11963623; doi:10.1093/bjsopen/zraf016)

**Sarcopenia, myosteatosis and frailty parameters to predict adverse outcomes in patients undergoing emergency laparotomy: a prospective observational multiple centre cohort study**

Brittany Park MBChB 1,2, Alain Vandal PhD 3, Fraser Welsh MBBS MSc FRACS 1, Tim Eglinton MBChB MMedSc FRACS FACS FCSSANZ 4, Jonathan Koea MD FACS FRACS 1, Ashish Taneja MBChB FRACS 1, 5, Ahmed Barazanchi MBChB FRACS 1, Andrew G. Hill MBChB MD FRACS FRSNZ 1,2, Andrew D. MacCormick MBChB PhD FRACS 1, 2.

1 Faculty of Medical and Health Sciences, The University of Auckland, Waipapa Taumata Rau, Auckland, Aotearoa New Zealand

2 Department of Surgery, Te Whatu Ora Counties Manukau, Auckland, Aotearoa New Zealand

3 Department of Statistics, The University of Auckland, Auckland, Aotearoa New Zealand

4 Faculty of Medical and Health Sciences, University of Otago, Aotearoa, New Zealand

5 Department of Surgery, Auckland City Hospital, Te Whatu Ora, Auckland, Aotearoa New Zealand

CORRESPONDING AUTHOR

Dr Brittany Park MBChB

Department of Surgery,

Faculty of Medical and Health Sciences, The University of Auckland, Waipapa Taumata Rau

Private Bag 93311, Otahuhu

Auckland, Aotearoa New Zealand

Email: Brittany.park09@gmail.com

Phone: (+64) 22 1541 780

**Supplementary Materials - Index**

| **Supplementary Figures and Tables** |  |
| --- | --- |
| Supplementary Table 1: Proportional Means (unbounded) Regression Analysis for hospital length of stay | *page 2* |
| Supplementary Table 2: Relative Risk Regression Analysis for 90-Day Mortality | *page 4* |
| Supplementary Table 3: Relative Risk Regression Analysis for 6-Month Mortality | *page 5* |
| Supplementary Table 4: Relative Risk Regression Analysis for Major Complications | *page 7* |
| Supplementary Figure 1: Directed Acyclic Graph for Primary Outcome: Rehabilitation | *page 9* |
| Supplementary Figure 2: Directed Acyclic Graph for Primary Outcome Days Alive and Out of Hospital | *page 10* |
| Supplementary Figure 3: Directed Acyclic Graph for Primary Outcome Risk of Not Returning Home | *page 11* |

**Supplementary Table 1: Proportional Means (unbounded) Regression Analysis for hospital length of stay**

| **Proportional Means Regression Analysis for hospital length of stay** | | | | | | |
| --- | --- | --- | --- | --- | --- | --- |
|  | **Simple Regression Analysis** | |  | **Multiple Regression Analysis** | | |
|  | **P.D** | **95% CI** | **p value** | **P.D** | **95% CI** | **p value** |
| **Combined Sarcopenia** | |  |  |  |  |  |
| *No* | Reference |  |  |  |  |  |
| *Yes* | +0.1% | -42.5,+85.7 | 0.998 | +3.4% | -33.0,+64.3 | 0.88 |
| **Low HGS** | |  |  |  |  |  |
| *No* | Reference |  |  |  |  |  |
| *Yes* | +14.5% | -31.1,+95.7 | 0.61 | +13.1% | -23.4,+69.3 | 0.53 |
| **Low SMI** | |  |  |  |  |  |
| *No* | Reference |  |  |  |  |  |
| *Yes* | -8.6% | -43.3,+46.0 | 0.71 | -6.7% | -37.2,+37.4 | 0.72 |
| **SARC-F Positive** |  |  |  |  |  |  |
| *No* | Reference |  |  |  |  |  |
| *Yes* | +51.2% | -14.0,+177.3 | 0.16 | +31.1% | -15.1,+108 | 0.23 |
| **Low SM-RA** | |  |  |  |  |  |
| *No* | Reference |  |  |  |  |  |
| *Yes* | +80.0% | +12.5,+194.1 | 0.019 | +38.2% | -9.7,+114 | 0.13 |
| **CFS≥5** |  |  |  |  |  |  |
| *No* | Reference |  |  |  |  |  |
| *Yes* | +80.0% | +10.3,+206.2 | 0.027 | +55.4% | +1.5,+143 | 0.038 |
| **Age** |  |  |  |  |  |  |
| *<80* | Reference |  |  |  |  |  |
| *≥80* | +15.4% | -35.9,+125.4 | 0.65 |  |  |  |
| **BMI** |  |  |  |  |  |  |
| *≥18.5,<25* | Reference |  |  |  |  |  |
| *≥25* | -18.4% | -50.1,+31.9 | 0.40 |  |  |  |
| *<18.5* | -29.7% | -64.0,+115.3 | 0.60 |  |  |  |
| **Diabetes** |  |  |  |  |  |  |
| *No* | Reference |  |  |  |  |  |
| *Yes* | -32.1% | -62.1,+32.7 | 0.23 |  |  |  |
| **Cardiopulmonary** | |  |  |  |  |  |
| *No* | Reference |  |  |  |  |  |
| *Yes* | +46.9% | -14.5,+169.3 | 0.19 |  |  |  |
| **Hypoalbuminemia** | |  |  |  |  |  |
| *No* | Reference |  |  |  |  |  |
| *Yes* | +32.3% | -19.1,+118.8 | 0.26 |  |  |  |
| **Cancer** |  |  |  |  |  |  |
| *No* | Reference |  |  |  |  |  |
| *Primary* | +0.9% | -49.0,+119.9 | 0.98 |  |  |  |
| *Disseminated* | +43.9% | -23.7,+205.9 | 0.30 |  |  |  |
| **ASA** |  |  |  |  |  |  |
| *ASA 1-2* | Reference |  |  |  |  |  |
| *ASA >2* | +113.3% | +38.5,+222.2 | <0.001 |  |  |  |
|  |  |  |  |  |  |  |

PD = percentage difference

*Proportional means unbounded regression analysis for hospital length of stay

**Supplementary Table 2: Relative Risk Regression Analysis for 90-Day Mortality**

| **Relative Risk Regression Analysis for 90-Day Mortality** | | | | | | |
| --- | --- | --- | --- | --- | --- | --- |
|  | **Simple Regression Analysis** | |  | **Multiple Regression Analysis** | | |
|  | **RR** | **95% CI** | **p value** | **RR** | **95% CI** | **p value** |
| **Combined Sarcopenia** | |  |  |  |  |  |
| *No* | Reference |  |  |  |  |  |
| *Yes* | 11.5 | 1.31, 101.2 | 0.028 | 8.65 | 1.13, 66.10 | 0.038 |
| **Low HGS** | |  |  |  |  |  |
| *No* | Reference |  |  |  |  |  |
| *Yes* | 6.90 | 0.77, 62.1 | 0.085 | 8.36 | 1.10, 63.50 | 0.040 |
| **Low SMI** | |  |  |  |  |  |
| *No* | Reference |  |  |  |  |  |
| *Yes* | 2.91 | 0.31, 26.9 | 0.35 | 1.52 | 0.18, 13.08 | 0.70 |
| **SARC-F Positive** |  |  |  |  |  |  |
| *No* | Reference |  |  |  |  |  |
| *Yes* | 4.48 | 0.38, 52.5 | 0.23 | 1.01 | 0.63, 162.40 | 0.10 |
| **Low SM-RA** | |  |  |  |  |  |
| *No* | Reference |  |  |  |  |  |
| *Yes* | 2.43 | 0.37, 16.0 | 0.36 | 2.11 | 0.33, 13.65 | 0.43 |
| **CFS≥5** |  |  |  |  |  |  |
| *No* | Reference |  |  |  |  |  |
| *Yes* | 1.35 | 0.15, 11.95 | 0.79 | 2.26 | 0.34, 15.16 | 0.40 |
| **Age** |  |  |  |  |  |  |
| *<80* | Reference |  |  |  |  |  |
| *≥80* | 4.05 | 0.62, 26.6 | 0.15 |  |  |  |
| **BMI** |  |  |  |  |  |  |
| *<25* | Reference |  |  |  |  |  |
| *≥25* | 0.71 | 0.11, 4.69 | 0.72 |  |  |  |
| *<18.5* | N/A |  |  |  |  |  |
| **Cardiopulmonary** |  |  |  |  |  |  |
| *No* | Reference |  |  |  |  |  |
| *Yes* | 3.84 | 0.59, 25.1 | 0.16 |  |  |  |
| **Hypoalbuminemia** |  |  |  |  |  |  |
| *No* | Reference |  |  |  |  |  |
| *Yes* | 5.82 | 0.63, 53.3 | 0.12 |  |  |  |
| **Cancer** |  |  |  |  |  |  |
| *No* | Reference |  |  |  |  |  |
| *Disseminated* | 1.91 | 0.21, 17.8 | 0.57 |  |  |  |
|  |  |  |  |  |  |  |

Excluded: Diabetes Mellitus, Primary cancer, ASA score. All outcome events in the non-diabetic group. All outco theme events in non-primary cancer group. All outcome events in ASA >2 group.

**Supplementary Table 3: Relative Risk Regression Analysis for Six-Month Mortality**

| **Relative Risk Regression Analysis for Six-Month Mortality** | | | | | | |
| --- | --- | --- | --- | --- | --- | --- |
|  | **Simple Regression Analysis** | |  | **Multiple Regression Analysis** | | |
|  | **RR** | **95% CI** | **p value** | **RR** | **95% CI** | **p value** |
| **Combined Sarcopenia** | |  |  |  |  |  |
| *No* | 1 |  |  | 1 |  |  |
| *Yes* | 5.70 | 1.10, 29.76 | 0.0393 | 4.52 | 1.04, 19.74 | 0.0448 |
| **Low HGS** | |  |  |  |  |  |
| *No* | 1 |  |  | 1 |  |  |
| *Yes* | 3.46 | 0.64, 18.68 | 0.1488 | 4.37 | 1.01, 18.88 | 0.0485 |
| **Low SMI** | |  |  |  |  |  |
| *No* | 1 |  |  | 1 |  |  |
| *Yes* | 3.99 | 0.46, 34.25 | 0.2077 | 1.58 | 0.19, 13.41 | 0.6760 |
| **SARC-F Positive** |  |  |  |  |  |  |
| *No* | 1 |  |  | 1 |  |  |
| *Yes* | 1.89 | 0.27, 13.26 | 0.5233 | 4.77 | 0.81, 28.05 | 0.0836 |
| **Low SM-RA** | |  |  |  |  |  |
| *No* | 1 |  |  | 1 |  |  |
| *Yes* | 1.65 | 0.30, 8.97 | 0.5646 | 2.08 | 0.35,12.49 | 0.4234 |
| **CFS≥5** |  |  |  |  |  |  |
| *No* | 1 |  |  | 1 |  |  |
| *Yes* | 1.04 | 0.13, 8.45 | 0.9700 | 1.89 | 0.29,12.24 | 0.5050 |
| **Age** |  |  |  |  |  |  |
| *<80* | 1 |  |  | 1 |  |  |
| *≥80* | 2.61 | 0.48, 14.29 | 0.2680 |  |  |  |
| **BMI** |  |  |  |  |  |  |
| *<25* | 1 |  |  | 1 |  |  |
| *≥25* | 0.46 | 0.08, 2.57 | 0.3799 |  |  |  |
| **Cardiopulmonary** |  |  |  |  |  |  |
| *No* | 1 |  |  | 1 |  |  |
| *Yes* | 2.51 | 0.46, 13.71 | 0.2880 |  |  |  |
| **Hypoalbuminemia** |  |  |  |  |  |  |
| *No* | 1 |  |  |  |  |  |
| *Yes* | 2.95 | 0.53, 16.26 | 0.2150 |  |  |  |
| **Cancer** |  |  |  |  |  |  |
| *No* | 1 |  |  | 1 |  |  |
| *Disseminated* | 3.79 | 0.70, 20.41 | 0.1210 |  |  |  |
| **ASA** |  |  |  |  |  |  |
| *ASA 1-2* | 1 |  |  |  |  |  |
| *ASA >2* | 1.37 | 0.16, 11.46 | 0.7730 |  |  |  |
|  |  |  |  |  |  |  |

Excluded: Diabetes mellitus, Primary cancer, All outcome events in non-diabetic group. All outcome events in group without primary cancer.

**Supplementary Table 4: Relative Risk Regression Analysis for Major Complications**

| **Relative Risk Regression Analysis for Major Complications** | | | | | | |
| --- | --- | --- | --- | --- | --- | --- |
|  | **Simple Regression Analysis** | |  | **Multiple Regression Analysis** | | |
|  | **RR** | **95% CI** | **p value** | **RR** | **95% CI** | **p value** |
| **Combined Sarcopenia** | |  |  |  |  |  |
| *No* | 1 |  |  |  |  |  |
| *Yes* | 0.71 | 0.29, 1.75 | 0.4576 | 0.48 | 0.21, 1.09 | 0.0807 |
| **Low HGS** | |  |  |  |  |  |
| *No* | 1 |  |  |  |  |  |
| *Yes* | 1.15 | 0.60, 2.23 | 0.6760 | 0.70 | 0.41,1.19 | 0.1913 |
| **Low SMI** | |  |  |  |  |  |
| *No* | 1 |  |  |  |  |  |
| *Yes* | 0.75 | 0.40, 1.42 | 0.3774 | 0.58 | 0.34, 0.98 | 0.0436 |
| **SARC-F Positive** |  |  |  |  |  |  |
| *No* | 1 |  |  |  |  |  |
| *Yes* | 0.93 | 0.40, 2.16 | 0.8603 | 0.80 | 0.46, 1.38 | 0.4195 |
| **Low SM-RA** | |  |  |  |  |  |
| *No* | 1 |  |  |  |  |  |
| *Yes* | 1.57 | 0.85, 2.91 | 0.1523 | 1.50 | 0.91, 2.47 | 0.1123 |
| **CFS≥5** |  |  |  |  |  |  |
| *No* | 1 |  |  |  |  |  |
| *Yes* | 1.26 | 0.62, 2.55 | 0.5289 | 1.40 | 0.91, 2.13 | 0.1233 |
| **Age** |  |  |  |  |  |  |
| *<80* | 1 |  |  |  |  |  |
| *≥80* | 1.44 | 0.73, 2.83 | 0.2904 |  |  |  |
| **BMI** |  |  |  |  |  |  |
| *<25* | 1 |  |  |  |  |  |
| *≥25* | 1.22 | 0.62, 2.38 | 0.5666 |  |  |  |
| *<18.5* | 0.51 | 0.08, 3.42 | 0.4860 |  |  |  |
| **Cardiopulmonary** |  |  |  |  |  |  |
| *No* | 1 |  |  |  |  |  |
| *Yes* | 0.65 | 0.10, 4.27 | 0.6560 |  |  |  |
| **Diabetes** |  |  |  |  |  |  |
| *No* | 1 |  |  |  |  |  |
| *Yes* | 0.74 | 0.11, 4.79 | 0.7500 |  |  |  |
| **Hypoalbuminemia** |  |  |  |  |  |  |
| *No* | 1 |  |  |  |  |  |
| *Yes* | 2.35 | 1.27, 4.36 | 0.0066 |  |  |  |
| **Cancer** |  |  |  |  |  |  |
| *No* | 1 |  |  |  |  |  |
| *Primary* | 1.27 | 0.50, 3.20 | 0.6110 |  |  |  |
| *Disseminated* | 1.51 | 0.72, 3.16 | 0.2749 |  |  |  |
| **ASA** |  |  |  |  |  |  |
| *ASA 1-2* | 1 |  |  |  |  |  |
| *ASA >2* | 2.37 | 0.88, 6.38 | 0.0871 |  |  |  |
|  |  |  |  |  |  |  |

**Supplementary Figure 1:**

Directed Acyclic Graph for Primary Outcome: Rehabilitation


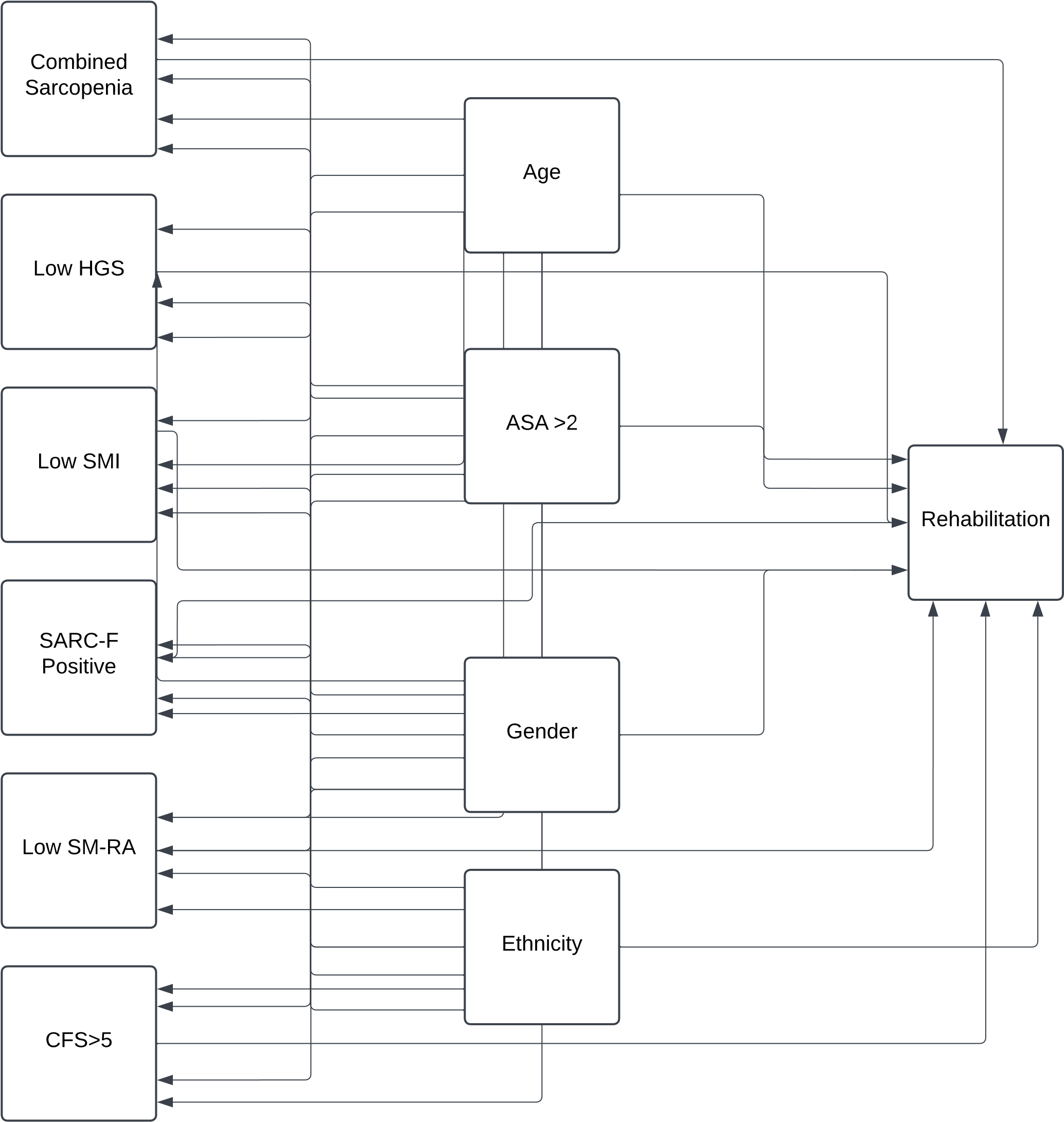


**Supplementary Figure 2:**

Directed Acyclic Graph for Primary Outcome Days Alive and Out of Hospital


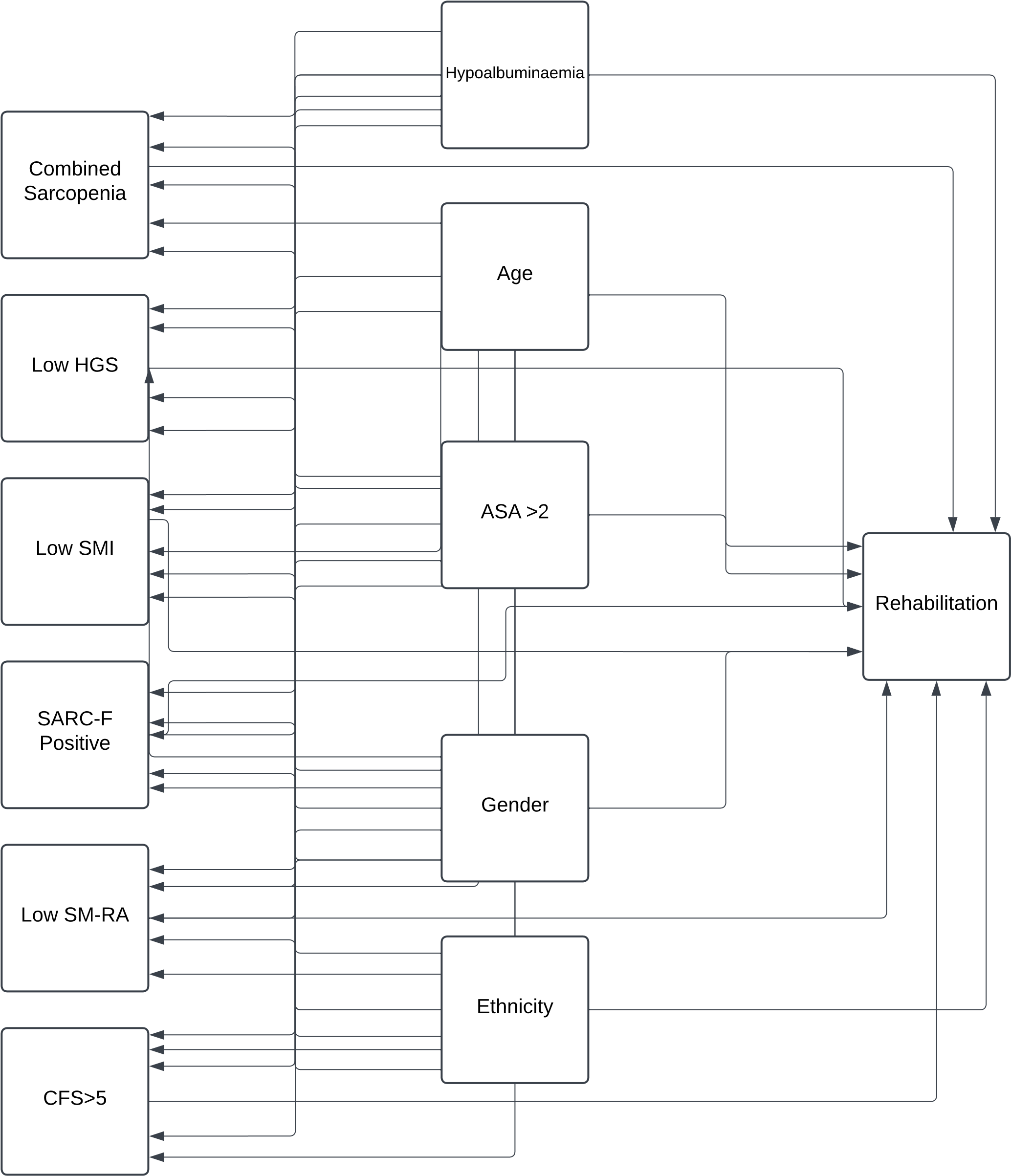


**Supplementary Figure 3:**

Directed Acyclic Graph for Primary Outcome Risk of Not Returning Home


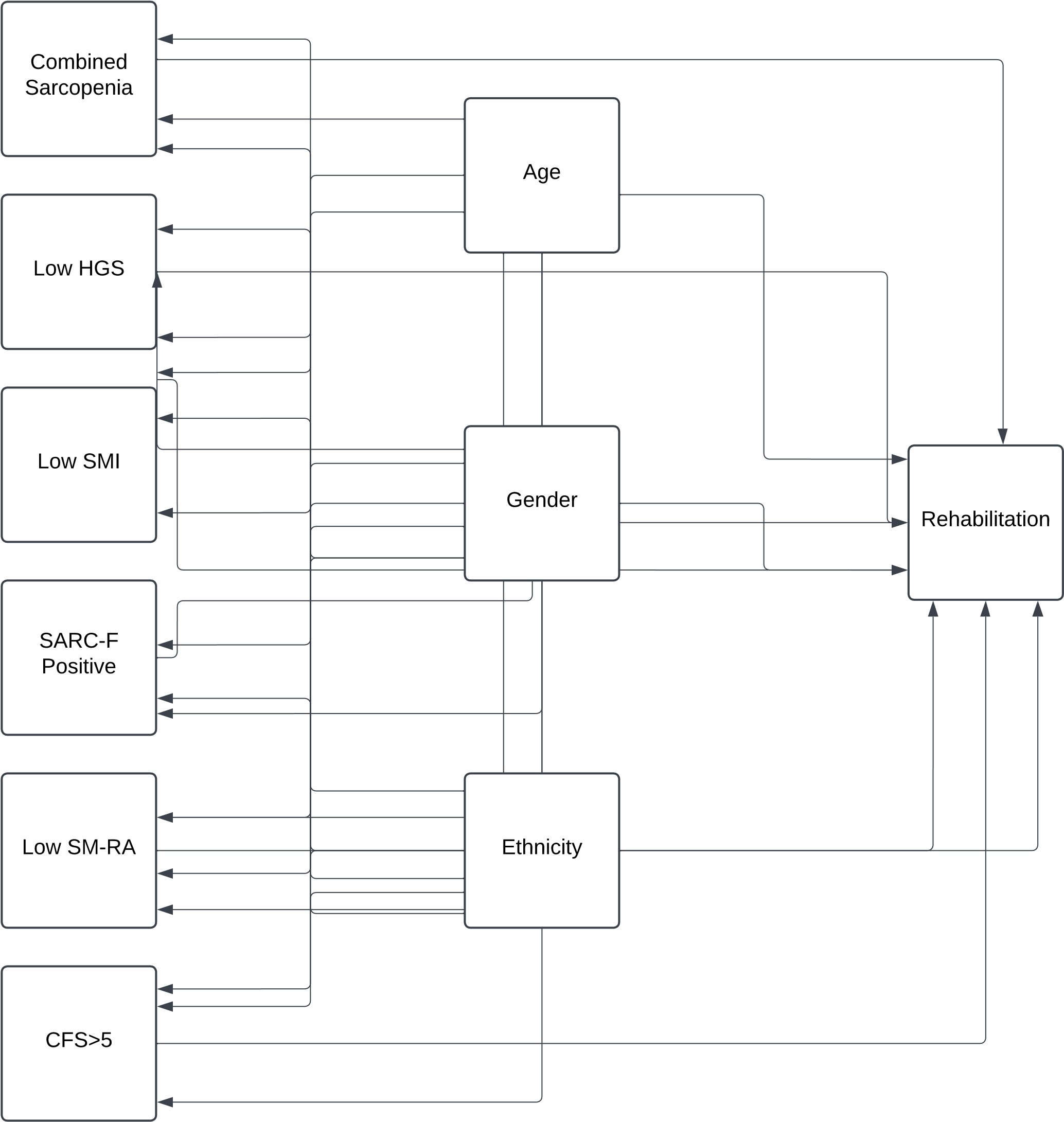

Supplement: zraf016_Supplementary_Data [file zraf016_supplementary_data.docx]
